# Outcomes of Pregnancy in COVID-19-Positive Mothers in a Tertiary Centre

**DOI:** 10.3390/life13071491

**Published:** 2023-06-30

**Authors:** Vigneshwaran Subramaniam, Beng Kwang Ng, Su Ee Phon, Hamizan Muhammad Rafi’uddin, Abd Razak Wira Sorfan, Abd Azman Siti Hajar, Mohamed Ismail Nor Azlin

**Affiliations:** Department of Obstetrics and Gynaecology, Faculty of Medicine, Universiti Kebangsaan Malaysia, Jalan Yaacob Latiff, Bandar Tun Razak, Cheras, Kuala Lumpur 56000, Malaysia; kinet246@gmail.com (V.S.); sephon88@yahoo.com (S.E.P.); rafinofee@gmail.com (H.M.R.); wirasorfan@gmail.com (A.R.W.S.); sitihajar.abdazman@gmail.com (A.A.S.H.); azlinm@ppukm.ukm.edu.my (M.I.N.A.)

**Keywords:** COVID-19, pregnancy, maternal, perinatal, infection, vaccination

## Abstract

Background: COVID-19 is an emerging global pandemic with potential adverse effects during pregnancy. This study aimed to determine the adverse maternal and foetal outcomes due to COVID-19 infection. We also compared maternal and neonatal outcomes with regard to the timing of diagnosis (first and second trimester vs. third and fourth trimester); early COVID-19 (stage I and II) vs. severe-stage COVID-19 (III, IV, and V); and lastly, women who were partially vaccinated vs. unvaccinated. Methods: This was a retrospective study conducted in HCTM from January 2021 to January 2022. All pregnant women admitted for COVID-19 infections were recruited. The patients’ records were traced. Adverse maternal and neonatal outcomes were documented and analysed. Results: There were 172 pregnant women recruited into this study. We excluded twenty-four patients with incomplete data and nine women who delivered elsewhere. The final 139 patients were available for data analysis. The majority of women were in their third trimester of pregnancy (87.8%); however, only 5.0% and 7.2% were in the first and second trimesters, respectively. The study population had a median BMI of 29.1 kg/m^2^ and almost half of them had never received a COVID-19 vaccination. A sub-analysis of data concerning adverse maternal and foetal outcomes comparing early vs. severe stages of COVID-19 infection showed that severe-stage disease increased the risk of preterm birth (54.5% vs. 15.4%, *p* < 0.001) and preterm birth before 34 weeks (31.9% vs. 2.6%, *p* < 0.001) significantly. The severe-stage disease also increased NICU admission (40.9% vs. 15.4%, *p* = 0.017) with lower birth weight (2995 g vs. 2770 g, *p* = 0.017). The unvaccinated mothers had an increased risk of preterm birth before 34 weeks and this was statistically significant (11.6% vs. 2.9%, *p* = 0.048). Conclusions: Adverse pregnancy outcomes such as ICU admission or patient death could occur; however, the clinical course of COVID-19 in most women was not severe and the infection did not significantly influence the pregnancy. The risk of preterm birth before 34 weeks was higher in a more severe-stage disease and unvaccinated mother. The findings from this study can guide and enhance antenatal counselling of women with COVID-19 infection, although they should be interpreted with caution in view of the very small number of included cases of patients in the first and second trimesters.

## 1. Introduction

The world was shaken in 2019 by the spread of an unknown virus causing pneumonia-like symptoms. Starting from a small city in Wuhan, China, the virus spread like wildfire and led to a global pandemic. The outbreak was caused by a novel coronavirus known as severe acute respiratory syndrome coronavirus-2 (SARS-CoV-2) and the disease is now named coronavirus disease 2019 (COVID-19) [1]. Due to the increasing mortalities from COVID-19 globally, the WHO announced this highly infectious disease as a global pandemic on 11th March 2020 [1]. Pregnant and non-pregnant women had the same risk of acquiring COVID-19 [1]. Due to physiologic, anatomic, and immunologic changes in pregnancy, there was an increased risk of complications during pregnancy and the management of pregnant women was more challenging and complicated. Pregnant women with COVID-19 were 5.4 times more likely to be hospitalised, 1.5 times more likely to be admitted to the ICU, and 1.7 times at higher risk of requiring mechanical ventilation [1].

Malaysia was not exempt from this global pandemic, with the first three imported cases confirmed on 25th January 2020. Since then, Malaysia has reported 2.75 million cases and 31,485 deaths as of 31 December 2021 [2]. The first large outbreak in Malaysia was managed successfully using movement restrictions between March and April 2020 [3]. Following the implementation of the MCO, all Malaysians were primarily instructed to stay indoors. Other imposed restrictions included a prohibition of mass gatherings, health screening and quarantine for Malaysians coming from abroad, restriction on foreigners entering the country, and closure of all facilities except primary and essential services such as health services, water, electricity, telecommunication, and food supply companies [3]. However, since September 2020, institutional outbreaks, state elections, and an inconsistent implementation of public health and social measures have led to large periodic outbreaks [4]. Hospital Canselor Tuanku Muhriz (HCTM) started seeing COVID-19 patients during pregnancy in January 2021.

Several studies conducted in Europe observed adverse pregnancy outcomes in COVID-19-infected women. Adverse pregnancy outcomes were associated with infection acquired at early gestational age, more symptomatic presentation, and use of oxygen support therapy. Maternal and neonatal outcomes include caesarean section, premature birth, low birth weight, COVID-19 transmission in neonates, maternal ICU admission, mechanical ventilation rates, and maternal death. Little evidence suggests that COVID-19 infection in early pregnancy causes preterm delivery and low birth weight in certain countries [5,6,7,8,9,10,11,12]. A recent systematic review regarding the effect of COVID-19 on maternal, perinatal, and neonatal outcomes among 324 pregnant women concluded that gestational age upon admission ranged from 5 weeks to 41 weeks, and up to 14% of women presented with severe pneumonia. There were four cases of spontaneous miscarriage, and gestational age upon delivery ranged from 28 weeks to 41 weeks. The majority of women delivered via caesarean section, and in those with severe disease, almost all required ICU admission [13].

Unfortunately, local data about the effect of COVID-19 infection on adverse maternal and neonatal outcomes are limited. This study will allow us to increase our knowledge about the effect of COVID-19 during pregnancy among the local population and help to redistribute resources in the management of COVID-19-related issues in the local setting. We aimed to determine the adverse maternal outcomes such as miscarriage, preterm birth, preterm prelabour rupture of membrane, SGA, IUGR, emergency caesarean section, post-partum haemorrhage, and maternal death. We also aimed to determine the adverse neonatal outcomes such as low birth weight, NICU admission, baby requiring mechanical intubation, neonatal COVID-19 infection, and neonatal death. In the sub-analysis, we also compared maternal and neonatal outcomes with regard to the timing of diagnosis (before 28 weeks vs. after 28 weeks); early COVID-19 (stage I and II) vs. severe-stage COVID-19 (III, IV, and V); and lastly, partially vaccinated women vs. unvaccinated women.

## 2. Materials and Methods

### 2.1. Study Design

This was a retrospective study conducted in HCTM from January 2021 to January 2022. All pregnant women admitted for COVID-19 infection were recruited. Patients’ records were traced from the record office unit, which includes the patient’s social characteristics (age, parity, gestational age upon admission, body mass index, and pre-existing antenatal co-morbidity (e.g., diabetes mellitus, hypertension, and hyperthyroidism)), their presenting complaint (asymptomatic, fever, cough, chest tightness, sore throat, SOB, diarrhoea, loss of smell, loss of taste, myalgia, fatigue, and headache), and laboratory testing performed during admission (TWC, lymphocyte count, CRP, ALT, and AST). Adverse maternal outcomes such as miscarriage, preterm birth, preterm prelabour rupture of membrane, SGA, IUGR, emergency caesarean section, post-partum haemorrhage, ICU admission, need for mechanical ventilation, antiviral therapy used, required antibiotic therapy, LMWH used, corticosteroid used, and maternal death were documented on the data collection sheet. Lastly, adverse neonatal outcomes such as low birth weight, poor Apgar score, NICU admission, mechanical intubation, oxygen support, neonatal COVID-19 infection, and neonatal death were also documented. 

The diagnosis of COVID-19 was based on the results of real-time reverse transcriptase polymerase chain reaction (rRT-PCR) or RTK Ag detection and positive saliva test. The samples were collected from upper respiratory nasopharyngeal swabs to confirm COVID-19 either from government hospitals or private hospitals. Patients’ medical records with incomplete data or who delivered elsewhere were excluded from the study. We included all of the pregnant women who were admitted to HCTM for COVID-19 infection and delivered in our hospital. Exclusion criteria were patients with incomplete data and who delivered elsewhere. 

### 2.2. Sample Size Calculation

This study reports the rates or prevalence of clinical manifestations (symptoms, laboratory, and radiological findings) and risk factors of maternal and perinatal outcomes (preterm birth, foetal distress, and low birth weight) in pregnancies of women with COVID-19 and admitted to HCTM. Following the formula calculator that Epitools used, we used a prevalence calculator similar to the prevalence used by Smith et al. [9]. Therefore, based on the calculated sample size, about 139 patients were needed for this study.

### 2.3. Data Analysis

The Statistical Package of Social Sciences (SPSS) Version 22.0 (IBM Corp., Armonk, NY, USA) was used to analyse the study data. Data that were not normally distributed were expressed as median (quartile). Other statistical tests included Mann–Whitney U and Fisher exact/chi-square tests. A probability value of <0.05 was considered to be statistically significant.

## 3. Results

There were 172 pregnant women recruited into this study. We excluded twenty-four patients with incomplete data and nine women who delivered elsewhere. The final 139 patients were available for data analysis. The median maternal age was 32 years old, Para two, and the median gestational age upon admission was 38 weeks gestation. The majority of women were in their third trimester of pregnancy (87.8%), with only 5.0% and 7.2% being in the first and second trimesters, respectively. The ethnicity distribution followed the Malaysian population with the majority of the women being Malay (87.1%), followed by others (7.2%), and then Chinese and Indian (2.9% each). More than half of the women were professional in occupation, followed by housewives (34.5%). Most women received tertiary education and belonged to the M40 group according to household income in Malaysia. The study population had a median BMI of 29.1 kg/m2 and almost half of them had never received a COVID-19 vaccination (Table 1).

Almost three quarters of patients diagnosed with COVID-19 were diagnosed using COVID-19 PCR testing. The majority presented in the early stages (stages 1 and 2), but 16.8% of patients were diagnosed with COVID-19 stage 3A and above. Sixty percent of them were symptomatic upon diagnosis. Symptoms such as cough, sore throat, and fever were among the most common symptoms experienced by patients. Other symptoms included myalgia (28.8%), fatigue (20.1%), headache (8.6%), loss of smell (23.3%), and loss of taste (28.8%). Almost 60% of patients were given LMWH for the prevention of pulmonary embolism, but only 13.6% were given steroids (Table 2).

Upon admission, laboratory testing was performed that included a median white blood count of 10.3, a lymphocyte count of 16.3, and a CRP of 2.1. Both median ALT and AST were 15.0 and 14.0, respectively. There were 107 chest radiographs performed: 6.5% revealed ground-glass opacity and 12.1% observed pneumonia changes. Only ten CT scans were performed, and only one patient was diagnosed with pulmonary embolism (Table 3).

With regard to pregnancy outcomes, there were five miscarriages (3.6%) and 22.4% of the pregnancies were delivered before 37 weeks. Upon further analysis, 7.5% of the patients delivered preterm before 34 weeks, with 1.4% of SGA and 2.9% of IUGR. Almost 60% of patients delivered via lower segment caesarean section (LSCS), followed by vaginal delivery (32.4%) and instrumental delivery (3.6%). Of the caesarean deliveries, 85.7% were emergency, and 14.3% were elective. The reasons for elective caesarean section included being COVID-19-positive (58.3%), followed by other obstetrics indications such as two previous scars, refused trial of scar, and placenta praevia. Almost half of the patients underwent emergency LSCS due to COVID-19 in labour, and only 5.3% of deliveries were complicated with PPH (Table 4).

The median birth weight was 2990 g and almost 20.1% were admitted to the NICU due to various reasons such as presumed sepsis (11.1%), respiratory distress (29.7%), and prematurity (59.3%). There were a total of 16 out of 27 babies admitted to the NICU who required oxygen support, half of the babies required mechanical ventilation, and the other half were given oxygen support such as CPAP or nasal oxygen. There were no neonatal deaths in our study population (Table 5).

There were no statistically significant differences when comparing the timing of diagnosis of COVID-19 infection in each trimester (first and second trimester and third trimester onward) with regard to maternal and foetal outcomes, except for the miscarriage (Table 6).

A sub-analysis of data concerning adverse maternal and foetal outcomes comparing early vs. severe stages of COVID-19 infection showed that severe-stage disease increased the risk of preterm birth (54.5% vs. 15,4%, *p* < 0.001) and preterm birth before 34 weeks (31.9% vs. 2.6%, *p* < 0.001) significantly. Severe-stage disease also increases NICU admission (40.9% vs. 15.4%, *p* = 0.017) and lower birth weight (2995 g vs. 2770 g, *p* = 0.017) (Table 7). 

Lastly, unvaccinated mothers had an increased risk of preterm birth before 34 weeks and this was statistically significant (11.6% vs. 2.9%, *p* = 0.048) (Table 8). 

## 4. Discussion

The COVID-19 pandemic has been the greatest communicable disease outbreak. Malaysia has experienced several waves of infection, leading to a devastated health system. Malaysia reported 2.75 million cases and 31,485 deaths as of 30 December 2021. The disease burden studied by Jayaraj et al. showed that approximately 32.8% of the total population in Malaysia was estimated to have been infected with COVID-19 by the end of December 2021 [14]. The proportion of COVID-19 infections in ages 0–11, 12–17, 18–50, 51–65, and above 65 years were 19.9% (*n* = 1,982,000), 2.4% (*n* = 236,000), 66.1% (*n* = 6,577,000), 9.1% (*n* = 901,000), and 2.6% (*n* = 256,000), respectively [14]. The Malaysian MOH, since the outset, prepared for the worst-case scenarios and outlined the plan in clear and easily accessible guidelines. The mitigation strategies that have been in place for disease containment have included movement control order (MCO), enhanced MCO, social distancing, flattening the epidemic curve, vaccination, and achieving herd immunity [15,16]. 

Pregnant women are susceptible to COVID-19 complications due to gestation-related physiological changes. A cross-sectional study conducted during the Malaysian MCO showed that the majority of women (95%) demonstrated an adequate level of knowledge on COVID-19, whilst 99% had good practice [17]. Women with adequate knowledge also reported a more positive perception of MCO and better obstetric care experience. Additionally, the author also found that tertiary education, employment status, and higher household income were independent predictors of adequate maternal knowledge of COVID-19. Younger and nulliparous women demonstrated greater anxiety levels [17]. Our study population was young women with a median age of 32 years old, Para two. More than half of the women were professional and received a tertiary level education, with 60% of them being M40 and above. 

Currently, the results of different studies on the asymptomatic proportion vary significantly from country to country, by at least 1.4% up to 80% [18,19]. On the other hand, among these COVID-19 patients who experience symptoms, about 80% of them develop mild to moderate symptoms. In comparison, 10–20% of cases present severe symptoms throughout the disease, and about 5–6% become critically ill with ARDS, multi-organ failure, and/or septic shock [20]. In our study, 15.8% of the women presented at least stage 3 of the disease and 60.4% were symptomatic. We had three maternal deaths due to COVID-19 among the study population. They were aged between 28 and 37 years old, and presented between 27 and 33 weeks. All three of them were obese, came in with stage 5 disease, and were admitted to the ICU. Two of them were not vaccinated at all and another one only received one dose of the vaccine. All of the babies were delivered before 34 weeks, with babies weighing between 700 and 1600 g. However, none of the babies were COVID-19-positive.

The implementation of lockdowns or MCO over a long period of time in several countries, including Malaysia, has caused an economic crisis, either at the individual or national level. The impact of the economic burden, rates of unemployment, loss of income, and increased mental health issues were found to increase steadily, especially among the young, women, and poor families [21]. Thus, constant lockdowns were not the best way to combat the spread of COVID-19. The Malaysian government established the National COVID-19 Immunisation Program (PICK), which served as a coping strategy or mechanism to increase the Malaysian population’s herd immunity in dealing with the COVID-19 hazard. This initiative aimed to vaccinate 80 percent (23.6 million) of Malaysia’s population by February 2022 [22]. Unfortunately, until mid-2022, the number of PICK registrations (particularly for booster doses) in Malaysia remained low and unsatisfactory due to some vaccine hesitancy [23,24]. 

In our study, almost half (47.8%) of the pregnant women remained unvaccinated. A total of 17.9% of women had received one dose, 32.8% had completed two doses of vaccine, and only two women had had their first booster dose. All five miscarriages in our study were from the unvaccinated group. We also found that the unvaccinated group had a higher risk of preterm birth before 34 weeks. This contradicts a study by Wainstock et al., in which there were no differences found between the groups in terms of pregnancy, delivery, and newborn complications, including gestational age at delivery, the incidence of SGA, and newborn respiratory complications [25]. However, we could not demonstrate any correlation between vaccination status vs. stages of COVID-19. This is in contrast with the more recently published systematic review and meta-analysis (2023), where the rate of COVID-19 infections among vaccinated pregnant women compared to unvaccinated is significantly reduced by 43% [12].

With regard to the effects of COVID-19 infection during pregnancy, adverse pregnancy outcomes were associated with infection acquired at early gestational age, severe COVID-19 stage, and use of oxygen support therapy [5,6,7,8,9,10,11,12]. In this study, we could not demonstrate the adverse pregnancy outcome if the patient presented earlier gestation, as the sample size for patients in the first trimester and second trimester was only 5.0% and 7.2%, respectively. This is probably due to the fact that quite a number of patients presented earlier at our institution and were discharged or home monitored, but never delivered in our centre. However, we managed to demonstrate that the presentation of COVID-19 at stage 3 and above (severe stage) was associated with preterm birth, preterm birth before 34 weeks, higher risk of NICU admission, and lower birth weight. 

Looking at the data from our neighbour, Singapore, where a prospective cohort study was performed among 16 patients, 37.5%, 43.8%, and 18.7% were infected in the first, second, and third trimesters, respectively. Two patients aged ≥35 years (12.5%) developed severe pneumonia; one patient (body mass index: 32.9 kg/m^2^) required transfer to intensive care. There were no maternal mortalities. Five pregnancies produced term live births, while two spontaneous miscarriages occurred at 11 and 23 weeks [26].

In the earlier systematic review and meta-analysis by Di Mascio D et al. (2019), which included nineteen studies with 79 hospitalised women, preterm birth (24.3%) was the most common adverse pregnancy outcome. COVID-19 infection was associated with a higher rate of PPROM (20.7%), preeclampsia (16.2%), IUGR (11.7%), caesarean section delivery (84%), and perinatal death (11.1%) [6]. In another systematic review by Di Toro F et al., which included 24 studies and 1100 pregnancies, the prevalence of pneumonia was 89%, and 8% of women were admitted to the ICU. Three stillbirths and five maternal deaths were reported. The prevalence of COVID-19-related admission to the neonatal intensive care unit was 2%. Nineteen out of four hundred and forty-four neonates were positive for COVID-19 at birth [7]. In our study, we had a total of three maternal deaths but no cases of neonatal death. There were five babies who were confirmed to be COVID-19-positive in our study. 

Lastly, the issue of vertical transmission of severe acute respiratory syndrome coronavirus 2 (SARS-CoV-2) is still not well established. In a review that involved fifty-one studies reporting 336 newborns screened for COVID-19, only 15 (4.4%) were positive for throat swab RT-PCR [27]. Among neonates who were SARS-CoV-2-positive after throat swab, only five (33.3%) had concomitant placenta, amniotic fluid, and cord blood samples tested, and of which, only one amniotic fluid sample was positive via RT PCR [27]. Thus, it is still debatable whether vertical transmission occurred during the first trimester of pregnancy. Additionally, there is no evidence to support caesarean delivery, abstaining from breastfeeding, or mother and infant separation [27,28]. 

The strength of this study is that this is probably the first local study looking at maternal and foetal outcomes due to COVID-19 infection during pregnancy with a relatively good sample size. Besides reporting the profile of patients and the clinical features, we also looked at the effect of early diagnosis, stages of COVID-19, and vaccination status on pregnancy outcomes. However, there were a few limitations in this study. We did not have enough samples or representative cases from the first and second trimesters; thus, the data on the outcomes of pregnancy should be analysed with caution. We lost the follow-up when the patient was diagnosed early but did not deliver in our centre. We could not assess and further analyse the placental involvement in all of these cases, as there was another project looking at placental histo-morphological patterns, disease severity, and perinatal outcomes.

## 5. Conclusions

Adverse pregnancy outcomes such as ICU admission or death could occur; however, the clinical course of COVID-19 in most women was not severe, and the infection did not significantly influence the pregnancy. The risk of preterm birth before 34 weeks was higher in those with more severe-stage disease and unvaccinated mothers. The findings from this study can guide and enhance antenatal counselling of women with COVID-19 infection, although they should be interpreted with caution in view of the very small number of cases in the first and second trimesters.

## Figures and Tables

**Table 1:** Maternal characteristics.

Maternal Characteristic	N (%)
Age (years)	32.0 (28.0, 35.0)
Parity	2 (1, 3)
Gestational age upon admission	38.0 (33.0, 39.1)
Trimester	
First	7 (5.0)
Second	10 (7.2)
Third	122 (87.8)
Ethnicity	
Malay	121 (87.1)
Chinese	4 (2.9)
Indian	4 (2.9)
Other (non-Malaysian)	10 (7.1)
Occupation	
Professional	72 (51.8)
Non-professional	19 (13.7)
Housewife	48 (34.5)
Educational level	
Primary	6 (4.3)
Secondary	48 (34.5)
Tertiary	80 (57.6)
No formal education	5 (3.6)
Household income	
B40 (<RM 4360)	55 (39.5)
M40 (>RM 4360–RM9619)	71 (51.1)
T20 (>RM9616)	13 (9.4)
Body mass index (kg/m^2^)	29.1 (25.5, 32.4)
Medical comorbid	37 (26.6)
Diabetes	13 (40.6)
Hypertension	8 (25.0)
Hyperthyroidism	2 (6.2)
Bronchial asthma	9 (28.1)
Status vaccination of mother	
Completed the first dose	24 (17.9)
Completed the second dose	44 (32.8)
Completed the first booster	2 (1.5)
Not vaccinated	64 (47.8)

Data presented as median (quartile) unless otherwise specified.

**Table 2:** Diagnosis and clinical presentation.

	N (%)
Type of test performed	
Saliva RTK antigen	16 (11.5)
Rapid mole PCR	16 (11.5)
PCR	107 (77.0)
COVID-19 category	
1	55 (39.6)
2A	39 (28.1)
2B	23 (16.5)
3A	5 (3.6)
3B	1 (0.7)
4A	6 (4.3)
4B	6 (4.3)
5	4 (2.9)
Clinical presentation	
Symptomatic	84 (60.4)
Fever	58 (41.7)
Cough	66 (47.5)
Chest tightness	9 (6.5)
Sore throat	64 (46.0)
Shortness of breath	20 (14.4)
Vomiting	17 (12.2)
Diarrhoea	21 (15.1)
Loss of smell	28 (20.1)
Loss of taste	32 (23.0)
Myalgia	40 (28.8)
Fatigue	28 (20.1)
Headache	12 (8.6)
Drug used	
Antiviral	9 (6.5)
Antibiotic	26 (18.7)
LMWH (enoxaparin)	83 (59.7)
Steroids	19 (13.7)

RTK: rapid test kit, PCR: polymerase chain reaction, LMWH: low-molecular-weight heparin.

**Table 3:** Laboratory testing and radiological imaging.

Laboratory Testing	N (%)
WBC (*n* = 139), ×10^9^/L	10.3 (7.9, 12.2)
Lymphocyte (*n* = 139), ×10^9^/L	16.3 (11.2, 21.9)
CRP (*n* = 139), mg/dL	2.1 (1.1, 3.8)
ALT (*n* = 139)	15.0 (11.0, 21.0)
AST (*n* = 61)	14.0 (11.0, 20.0)
Chest radiograph (*n* = 107)	
Ground-glass opacity	7 (6.5)
Observed pneumonia changes	13 (12.1)
Clear CXR	87 (81.3)
CT thorax (*n* = 10)	
PE	1 (10)
No PE	9 (90)

WBC: white blood count, CRP: C-reactive protein, AST: Aspartate Aminotransferase, ALT: Alanine Transaminase, PE: pulmonary embolism. Data presented as median (quartile) unless otherwise specified.

**Table 4:** Pregnancy outcomes.

Pregnancy Outcome	N (%)
Miscarriage	5 (3.6)
Preterm birth less than 37 weeks	30 (22.4)
Preterm birth less than 34 weeks	10 (7.5)
Preterm prelabour rupture of membrane	3 (2.2)
SGA	2 (1.4)
IUGR	4 (2.9)
Mode of delivery	
SVD	45 (32.4)
Instrumental delivery	5 (3.6)
LSCS	84 (60.4)
Nature of LSCS	
Elective	12 (14.3)
Emergency	72 (85.7)
Reason for elective LSCS	
COVID-19-positive	7 (58.3)
Two previous scars	2 (16.7)
Refused VBAC	2 (16.7)
Placenta praevia major	1 (8.3)
Reason for emergency LSCS	
COVID-19-positive in labour	34 (47.2)
Foetal distress	19 (26.4)
Poor progress	1 (1.4)
Maternal indication	9 (12.5)
Abnormal lie in labour	8 (11.1)
Bleeding placenta praevia	1 (1.4)
Post-partum complication	
PPH	7 (5.3)

SGA: small for gestational age; IUGR: intrauterine growth restriction; SVD: spontaneous vertex delivery; LSCS: lower segment caesarean section; VBAC: vaginal delivery after caesarean; PPH: post-partum haemorrhage; maternal indications include a higher stage of COVID-19, e.g., stage 4B and stage 5.

**Table 5:** Neonatal outcomes.

Neonatal Outcome	N (%)
Birth weight (gram)	2990 (2547, 3282)
NICU admission	27 (20.1)
Reason for admission, *n* = 27	
Presumed sepsis	3 (11.1)
Respiratory distress	8 (29.6)
Prematurity	18 (59.3)
Oxygen support, *n* = 16	
Mechanical ventilation	8 (50.0)
Oxygen other than mechanical ventilation	8 (50.0)
Baby COVID-19-positive, *n* = 134	5 (3.7)
Neonatal death	0

Data presented as median (quartile) unless otherwise specified.

**Table 6:** Comparison of maternal and neonatal outcomes based on the diagnosis of COVID-19.

Pregnancy Outcome	First and Second Trimester *n* = 16	Third Trimester Onward *n* = 123	*p* (Value)
Miscarriage, *n* (%)	5 (31.3)	0	<0.001
Preterm birth, *n* (%)	4 (26)	20 (16.2)	0.109
Preterm birth less than 34 weeks, *n* (%)	2 (12.5)	8 (6.5)	0.192
Preterm prelabour rupture of membrane, *n* (%)	0	3 (2.4)	1.000
SGA, *n* (%)	0	3 (2.4)	1.000
IUGR, *n* (%)	0	4 (3.3)	1.000
Mode of delivery, *n* (%)			
SVD Instrumental delivery LSCS	6 (37.5) 0 5 (31.3)	39 (31.7) 5 (4.1) 79 (64.2)	0.275
Post-partum complication			
PPH, *n* (%)	1 (6.3)	6 (4.9)	0.459
Neonatal outcome			
Baby weight (gram)	2900 (2400, 3160)	2990 (2560, 3320)	0.305
NICU admission, *n* (%)	3 (18.8)	24 (19.5)	0.694
Mechanical intubation, *n* (%)	2 (12.5)	6 (4.9)	0.467
COVID-19-positive, *n* (%)	0	5 (4.1)	1.000

Data presented as median (quartile) unless otherwise specified.

**Table 7:** Comparison of maternal and neonatal outcomes based on the diagnosis of COVID-19 in early vs. severe stage.

Pregnancy Outcome	Early Stage (I, II) *n* = 117	Severe Stage (III, IV, V) *n* = 22	*p* (Value)
Miscarriage, *n* (%)	5 (4.3)	0	1.000
Preterm birth, *n* (%)	18 (15.4)	12 (54.5)	<0.001
Preterm birth less than 34 weeks, *n* (%)	3 (2.6)	7 (31.9)	<0.001
Preterm prelabour rupture of membrane, *n* (%)	1 (0.9)	2 (9.1)	0.070
SGA, *n* (%)	3 (2.6)	0	1.000
IUGR, *n* (%)	3 (2.6)	1 (4.5)	0.516
Mode of delivery, *n* (%)			
SVD Instrumental delivery LSCS	38 (32.5) 5 (4.3) 69 (59.0)	7 (31.8) 0 15 (68.2)	0.567
Post-partum complication			
PPH, *n* (%)	7 (6.0)	0	0.599
Neonatal outcome			
Baby weight (gram)	2995 (2602, 3342)	2700 (2027, 3210)	0.047
NICU admission, *n* (%)	18(15.4)	9 (40.9)	0.017
Mechanical intubation, *n* (%)	4 (3.4)	4 (18.2)	1.000
COVID-19-positive, *n* (%)	5 (4.3)	0	0.591

Data presented as median (quartile) unless otherwise specified.

**Table 8:** Comparison of maternal and neonatal outcomes based on the diagnosis of COVID-19 according to vaccination stage.

Pregnancy Outcome	At Least Partially Vaccinated *n* = 70	Unvaccinated *n* = 69	*p* (Value)
Miscarriage, *n* (%)	0	5 (7.2)	0.028
Preterm birth, *n* (%)	11 (15.7)	19 (27.5)	0.053
Preterm birth less than 34 weeks, *n* (%)	2 (2.9)	8 (11.6)	0.048
Preterm prelabour rupture of membrane, *n* (%)	1 (1.4)	2 (2.9)	0.606
SGA, *n* (%)	3 (4.3)	0	0.246
IUGR, *n* (%)	2 (2.8)	2 (2.9)	1.000
Mode of delivery, *n* (%)			
SVD Instrumental delivery LSCS	22 (31.4) 3 (4.3) 45 (64.2)	23 (37.7) 2 (2.9) 39 (56.5)	0.826
Post-partum complication			
PPH, *n* (%)	5 (7.1)	2 (2.9)	0.444
Neonatal outcome			
Baby weight (gram)	3100 (2607, 3352)	2885 (2525, 3200)	0.059
NICU admission, *n* (%)	12 (17.1)	15 (21.7)	0.395
Mechanical intubation, *n* (%)	3 (4.3)	5 (7.2)	1.000
COVID-19-positive, *n* (%)	2 (2.8)	3 (4.3)	0.669

Data presented as median (quartile) unless otherwise specified.

## Data Availability

The data presented in this study are available upon request from the corresponding author. The data are not publicly available due to ownership belonging to the institution where the study was conducted.
